# Occlusion enhanced pan-cancer classification via deep learning

**DOI:** 10.1186/s12859-024-05870-y

**Published:** 2024-08-08

**Authors:** Xing Zhao, Zigui Chen, Huating Wang, Hao Sun

**Affiliations:** 1 Department of Orthopaedics and Traumatology, The Chinese University of Hong Kong, Hong Kong, People’s Republic of China; 2 Warshel Institute for Computational Biology, The Chinese University of Hong Kong, Shenzhen, Guangdong People’s Republic of China; 3 Department of Microbiology, The Chinese University of Hong Kong, Hong Kong, People’s Republic of China; 4 Department of Orthopaedics and Traumatology, Li Ka Shing Institute of Health Sciences, The Chinese University of Hong Kong, Hong Kong, People’s Republic of China; 5 Department of Chemical Pathology, Li Ka Shing Institute of Health Sciences, The Chinese University of Hong Kong, Hong Kong, People’s Republic of China

**Keywords:** Pan-cancer classification, Marker gene identification, Deep neural network, Long short term memory, Occlusion

## Abstract

Quantitative measurement of RNA expression levels through RNA-Seq is an ideal replacement for conventional cancer diagnosis via microscope examination. Currently, cancer-related RNA-Seq studies focus on two aspects: classifying the status and tissue of origin of a sample and discovering marker genes. Existing studies typically identify marker genes by statistically comparing healthy and cancer samples. However, this approach overlooks marker genes with low expression level differences and may be influenced by experimental results. This paper introduces “GENESO,” a novel framework for pan-cancer classification and marker gene discovery using the occlusion method in conjunction with deep learning. we first trained a baseline deep LSTM neural network capable of distinguishing the origins and statuses of samples utilizing RNA-Seq data. Then, we propose a novel marker gene discovery method called “Symmetrical Occlusion (SO)”. It collaborates with the baseline LSTM network, mimicking the “gain of function” and “loss of function” of genes to evaluate their importance in pan-cancer classification quantitatively. By identifying the genes of utmost importance, we then isolate them to train new neural networks, resulting in higher-performance LSTM models that utilize only a reduced set of highly relevant genes. The baseline neural network achieves an impressive validation accuracy of 96.59% in pan-cancer classification. With the help of SO, the accuracy of the second network reaches 98.30%, while using 67% fewer genes. Notably, our method excels in identifying marker genes that are not differentially expressed. Moreover, we assessed the feasibility of our method using single-cell RNA-Seq data, employing known marker genes as a validation test.

**Supplementary Information:**

The online version contains supplementary material available at 10.1186/s12859-024-05870-y.

## Introduction

Cancer continues to be a major global health challenge and a leading cause of mortality, driving extensive research efforts to advance diagnosis and treatment techniques [1]. Cancer detection aims to accurately classify the tumor types within a sample, even in the early stages of the disease, and to identify specific markers for each cancer type. However, conventional clinical methods for cancer detection, relying on manual microscopic examination of morphological tumor characteristics, are labor-intensive, require extensive training, and are prone to human error [2].

In comparison, pan-cancer classification using Next-Generation Sequencing (NGS) holds great clinical potential in transcending traditional cancer diagnosis methods. It can not only contribute to the adoption of personalized medicine but may also reveal undiscovered connections between tumor types

Pan-cancer Classification methods that analyze Differentially Expressed Genes (DEGs) and use statistical algorithms with NGS data may fail to capture the interactions among genes that exhibit subtle variations in expression levels [3]. Beyond DEG analysis, several studies have adopted machine learning approaches, including k-Nearest Neighbor (KNN), Random Forest (RF), Support Vector Machine (SVM), and Multi-task learning, for pan-cancer classification [4–6]. These studies also utilize gene screening methods such as ANOVA tests, Principal Component Analysis (PCA), and autoencoders. However, there remains a significant challenge in the interpretability of marker gene selection, particularly in understanding and quantifying the impact of individual genes across different classes of pan-cancer classifications.

This paper introduces a novel framework, “GENESO,” designed for pan-cancer classification and marker gene discovery. GENESO utilizes advanced deep learning techniques, particularly the innovative symmetrical occlusion method, to analyze NGS RNASeq data from clinical samples. By employing symmetrical occlusion, GENESO not only predicts the status and tissue-of-origin of the samples but also assesses the significance of each gene in the classification process.

## Method

### Dataset preprocessing

As tumors can vary significantly, with different cells within the same tumor or among tumors of the same type having distinct characteristics, we gathered RNA-Seq data from various public sources to ensure a comprehensive dataset (detailed in Supplementary File 1). This dataset includes paired tumor and normal samples, along with some rare tumors. For example, Petrini et al.’s dataset comprises 286 thymus tumor samples from patients with diverse characteristics [7]. Additionally, RNA-Seq data on rare tumors such as paraganglioma and sarcoma were collected from Snezhkina et al. and Lesluyes et al., respectively [8, 9].

To organize the dataset, we categorized the RNA-Seq data based on the tissue of origin and further divided it according to the status of each sample (e.g., cancer, normal), resulting in 28 classes. We chose not to create finer categories to ensure sufficient samples in each class.

The RNA-Seq data was aligned against the human reference genome GRCh.38 using “Bowtie2”, “featurecounts” was used to quantify the number of mapped reads for each gene. Genes on sex chromosomes without coverage across all classes were excluded from analysis [10, 11]. No batch effect correction was applied to avoid introducing bias.

### Gene expression level normalization

Various methods have been employed for the normalization of read count data in previous studies, with the most common approaches being “Transcripts Per Kilobase Million” (TPM) and “Reads Per Kilobase Million” (RPKM). These methods are designed to mitigate the bias introduced by gene length by taking transcript length into account and performing a multiplication of a constant on the result as shown in Eq. 1.

1 $$\begin{aligned} & RPKM = 10^{9} \times \frac{{{\text{Reads mapped to gene}}}}{{{\text{Total reads}} \times {\text{Gene length}}}} \\ & TPM = 10^{6} \times \frac{{\frac{{{\text{Reads mapped}}}}{{{\text{Gene length}}}}}}{{\sum {\frac{{{\text{Reads mapped}}}}{{{\text{Gene length}}}}} }} \\ & NRC = \frac{{{\text{Reads mapped}}}}{{{\text{Total mapped reads}}}} \\ \end{aligned}$$

However, neither TPM nor RPKM are suitable for pan-cancer classification using neural networks. Instead, we employ “Normalized Counts” (NRC), which eliminates the constant number and gene length from the normalization process. There are three reasons why NRC is more suitable for neural network processing:

1. *Redundant constant number*: The Z-score normalization process within the neural network renders constants in the equations of TPM and RPKM unnecessary.
2. *Suitability for cross-sample comparisons*: Extensive research has shown that TPM and RPKM are more appropriate for comparing transcript expression within a single sample rather than facilitating cross-sample comparisons [12–15].
3. *Gene length influence in neural network*: During neural network prediction, genes are compared across multiple samples. Consequently, the influence of gene length remains consistent throughout all samples, enabling us to remove it without compromising classification performance.

### Neural network architecture

The LSTM (Long Short-Term Memory), as depicted in Eq. 2, serves as the fundamental unit of the LSTM layer. It incorporates three inputs and produces two outputs: the sequence input $x_t$, the cell state input from the previous LSTM cell $c_{t-1}$, the hidden state input from the previous LSTM cell $h_{t-1}$, the hidden state output $h_t$ and the cell state output $c_t$. In simple terms, the hidden state $h_t$ retains short-term memory, while the cell state $c_t$ preserves long-term or global memory.

2 $$\begin{aligned} & i_{t} = \sigma (W_{x} ix_{t} + W_{h} ih_{{t - 1}} + W_{c} ic_{{t - 1}} + b_{i} ) \\ & f_{t} = \sigma (W_{x} fx_{t} + W_{h} fh_{{t - 1}} + W_{c} fc_{{t - 1}} + b_{f} ) \\ & c_{t} = f_{t} c_{{t - 1}} + i_{t} \tanh (W_{x} cx_{t} + W_{h} ch_{{t - 1}} + b_{c} ) \\ & o_{t} = \sigma (W_{x} ox_{t} + W_{h} oh_{{t - 1}} + W_{c} oc_{t} + b_{0} ) \\ & h_{t} = o_{t} \tanh (c_{t} ) \\ \end{aligned}$$

**Fig. 1:**
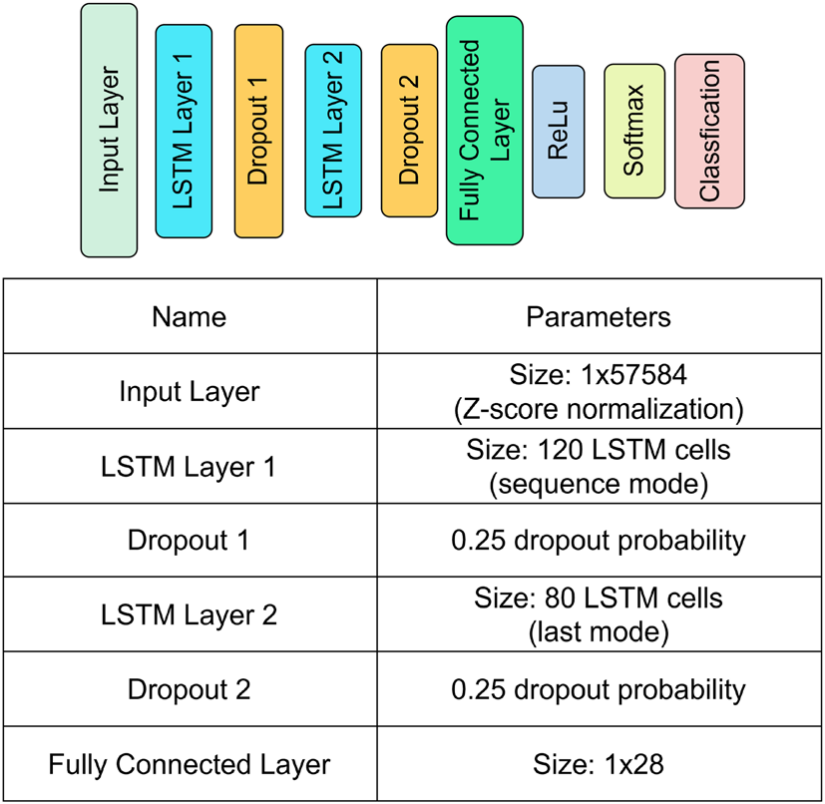
Overview of neural network architecture

As illustrated in Fig. 1, the initial layer of the neural network serves as the input layer, performing Z-score normalization on the input data. This normalization step is critical for achieving optimal network performance, enabling unbiased comparison of gene expression levels across samples regardless of scale differences.

Following the input layer, the neural network comprises two LSTM layers: the first with 120 cells and the second with 80 cells. This architecture allows the first layer to abstract normalized inputs, extract high-level information, and outperform convolutional neural networks with multiple convolutional layers [16].

To prevent overfitting and facilitate the identification of general patterns in the dataset, a dropout layer with a dropout probability of 30% is applied after each LSTM layer [17].

At the end of the neural network, a “Fully Connected” (FC) layer connects the LSTM layer and the output layer, generating predicted labels. The softmax and classification layer then produce the prediction labels based on the FC layer output, identifying the label with the highest prediction score.

### Marker gene identification

#### Overview

To identify marker genes and quantitatively evaluate their significance for pan-cancer classification, a novel algorithm named “Symmetrical Occlusion” (SO) is introduced. This method draws inspiration from “CNN occlusion” (CO), a feature identification technique used in neural network-based image classification, but it overcomes the limitations of CO in identifying signature genes.

SO assesses the significance of individual genes in pan-cancer classification using a neural network. It operates by mimicking both the “gain of function” and “loss of function” of genes. First, SO manipulates gene expression levels relative to the original values to create pseudo samples. Then, these pseudo samples are input into baseline neural networks to observe changes in the network’s output. These fluctuations are then used to quantify the importance of individual genes.

#### CNN occlusion method and its drawbacks

The CO method is widely used for identifying important regions within an image. The theory behind CO is that blocking or occluding a crucial region typically results in a sharp decrease in the prediction score, indicating the probability of the input image belonging to the corresponding class [18, 19]. This change can rapidly and accurately identify important regions for the neural network.

However, directly applying CO to marker gene identification faces challenges due to disparities in data structure between images and gene expression data. Unlike images which is a two-dimensional matrix, gene expression data is represented as a vector based on gene position, lacking the inherent spatial relationships present in pixel data. Moreover, gene expression ranges vary between samples, in contrast to the fixed pixel value range in images.

#### Symmetrical occlusion

##### Training of baseline neural network

**Fig. 2:**
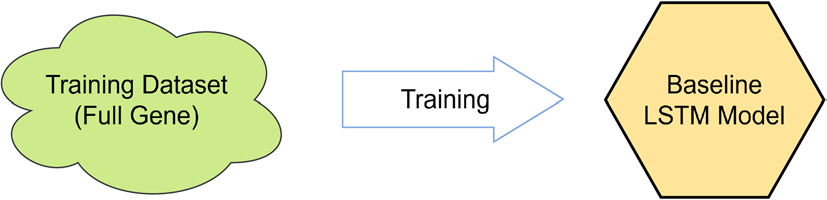
A baseline LSTM model is trained first for the occlusion

The SO method employs a multi-step process to assess the importance of a specific gene in pan-cancer classification. Initially, a baseline LSTM neural network is trained using the entire dataset of genes (Fig. 2). This step establishes the baseline performance for pan-cancer classification which is essential for subsequent comparisons.

##### “Gain of function” and “loss of function” simulation

Next, the importance of a specific gene in pan-cancer classification is quantified using SO by simulating its **“gain of function”** and **“loss of function”** effects.

In the “gain of function” simulation, the expression level of a gene in reference samples from the validation dataset is systematically occluded or replaced with increasing “occlusion values” in a step-wise manner, with each step being one-tenth of the original gene expression level. This process generates new pseudo samples with the gene replaced by new expression levels until the expression level exceeds twice the maximum value of the gene with the highest expression level.

Similarly, the “loss of function” simulation involves replacing the gene’s expression level with lower occlusion values than reference samples until the expression level reaches zero. This procedure is repeated for all reference samples across the 28 classes to enhance robustness. While it is biologically impossible for the gene to reach certain expression levels in some of the pseudo samples, the existence of these “imaginary” pseudo samples is a vital part of the simulation process.

**Fig. 3:**
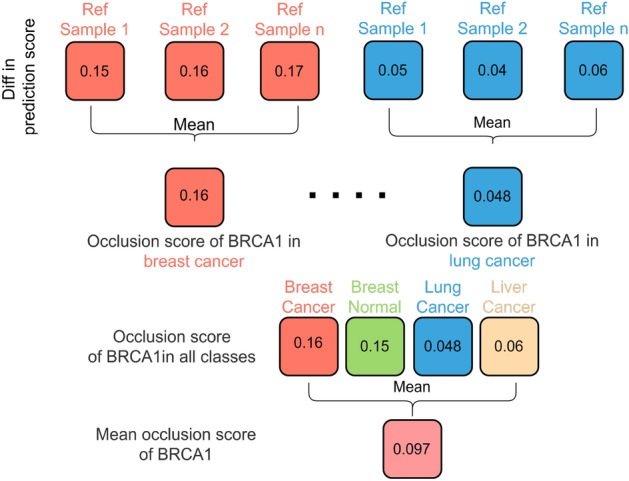
Generation of pseudo samples from reference sample using symmetrical occlusion. The biologically-impossible pseudo samples are categorized as “imaginary” pseudo samples. The gene expression level in this figure are fictional

For example, as illustrated in Fig. 3, during the “gain of function” simulation of gene BRCA1 on a sample where its expression level is 10 and the maximum expression level in this sample is 20, “gain of function” simulation would generate pseudo samples with BRCA1’s expression level being replaced by occlusion values ranging from 10.1 to 40. Likewise, “loss of function” would yield pseudo samples with decreasing occlusion values. Among these pseudo samples, those with biologically impossible gene expression levels are defined as “imaginary” pseudo samples, while the rest are categorized as “real” pseudo samples.

##### Quantification of gene importance

Pseudo samples are input into the baseline LSTM neural network which outputs prediction scores measuring the confidence of the neural network in classifying the samples. By combining the results from the “gain of function” and “loss of function” simulations, the influence of the occlusion value on prediction scores for all 28 classes can be visualized through a series of line plots.

The occlusion score, indicating the importance of a gene in a specific class, is determined by calculating the absolute difference between the maximum and minimum prediction scores for the corresponding class across pseudo samples generated from the same reference sample. Subsequently, the occlusion score is obtained by averaging the absolute values obtained from all reference samples. Finally, the mean occlusion score of a gene across all classes is calculated, signifying its importance in pan-cancer classification.

**Fig. 4:**
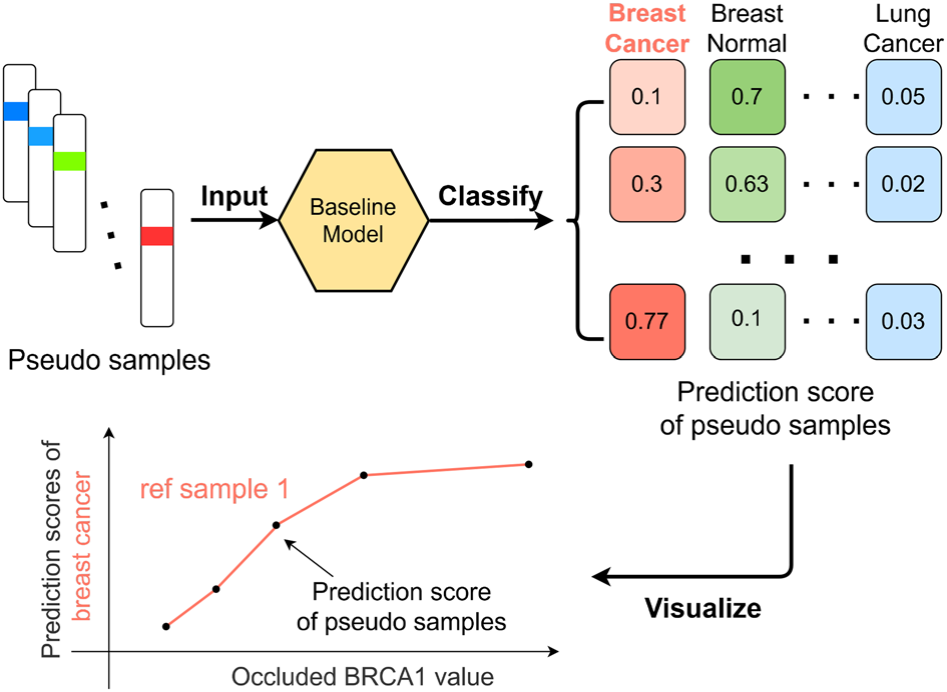
Visualization of the influence of symmetrical occlusion on gene BRCA1 by classifying the pseudo samples derived from reference sample using the baseline model and plotting the change in corresponding prediction score

Continuing the previous example, as illustrated in Fig. 4, a collection of prediction scores is obtained after passing the pseudo samples into the baseline neural network. For the “gain of function” simulation, an increase in the prediction score for the “breast cancer” class and a decrease in other classes such as “breast normal” is expected. After combining the results from “loss of function” simulation, the influence of occlusion value of gene BRCA1 on the reference sample can be visualized in a line plot.

**Fig. 5:**
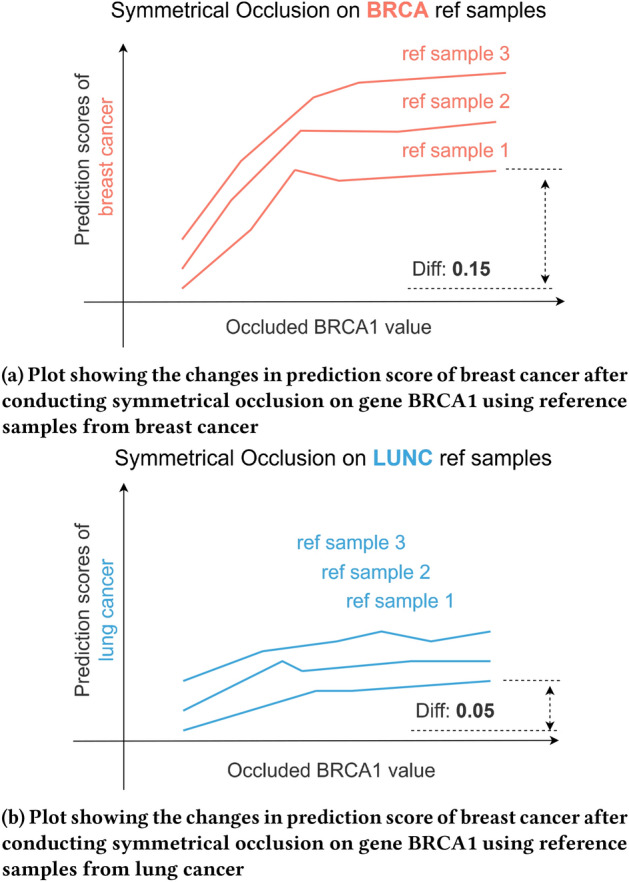
Comparison of prediction score differences in breast cancer (BRCA) and lung cancer (LUNC) after conducting symmetrical occlusion on gene BRCA1 in reference samples from corresponding classes. Higher differences in the prediction score suggest that gene BRCA1 is a more important gene in breast cancer

Repeating the step on the reference samples from the remaining 27 classes, the influence of gene BRCA1 on prediction scores on reference samples for all 28 classes can be visualized through a series of plots. As shown in Fig. 5a, gene BRCA1 exhibits great influence in the prediction score of the “breast cancer” class, resulting in an absolute difference between the maximum and minimum of 0.15 for breast cancer in one of the reference sample. It is higher than the absolute difference of 0.05 in one of the reference samples in lung cancer as shown in Fig. 5b. This demonstrates that gene BRCA1 is a more important gene in breast cancer than in lung cancer.

**Fig. 6:**
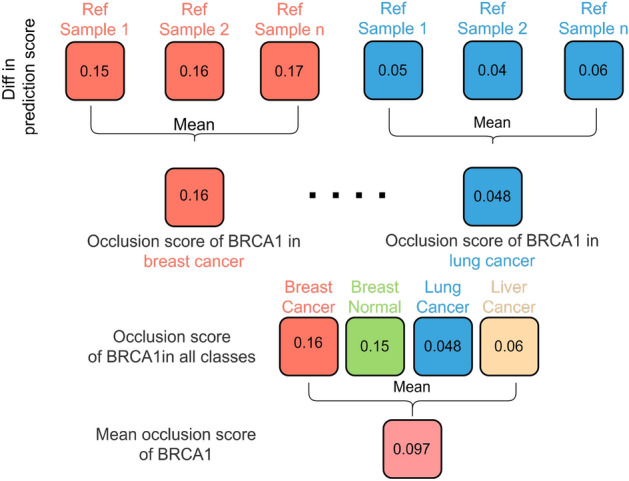
Calculation of occlusion score and the mean occlusion score of gene BRCA1

To enhance the robustness of the occlusion score, the difference between the maximum and minimum prediction scores from multiple reference samples is collected, and the mean is calculated and marked as the occlusion score. As illustrated in Fig. 6, the occlusion score of gene BRCA1 in breast cancer is 0.16, while its occlusion score in lung cancer is 0.048. By combining the occlusion scores of BRCA1 in other classes, the mean occlusion score of BRCA1, which measures its importance in pan-cancer classification, is 0.097.

#### Neural network optimization

**Fig. 7:**
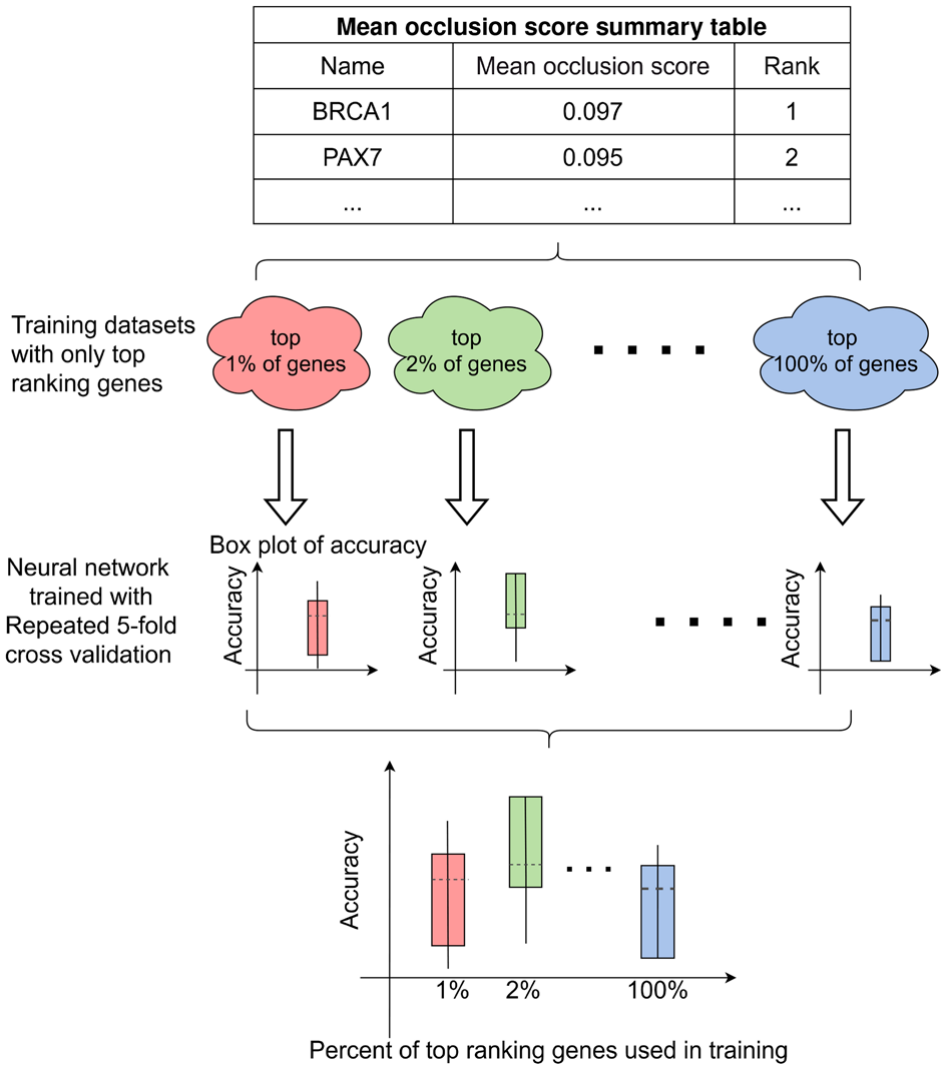
Training of new neural networks using training dataset containing different combination of genes ranked by their mean occlusion score to optimize the performance of the neural network

As illustrated in Fig. 7, after completing the steps in the previous section, a table summarizing the mean occlusion score of all genes is obtained. To enhance the pan-cancer classification accuracy of the LSTM neural network while using fewer genes, the genes in the summary table are initially ranked based on their mean occlusion score. Subsequently, new training datasets are generated by selecting different subsets of the top-ranking genes.

Using repeated fivefold cross-validation, new LSTM neural networks are trained on these datasets. The network exhibiting the highest validation accuracy is chosen as the final neural network. This approach facilitates the identification of a smaller subset of genes capable of achieving high classification accuracy, thereby improving efficiency and cost-effectiveness for future studies.

## Result

### Overview

In this study, we developed an LSTM neural network for pan-cancer classification and introduced the “Symmetrical Occlusion” algorithm to identify pivotal genes in classification while enhancing network performance with fewer genes. Our dataset comprised 3,524 samples classified into 28 classes, including 22 pairs of tumor and normal samples from the same tissue of origin. Through fivefold cross-validation, the network achieved an accuracy of 96.59% using NRC as a normalization method with the complete gene set. Conversely, the accuracy dropped to 91.84% and 89.53% when utilizing the complete gene sets of TPM and RPKM, respectively, indicating the superiority of NRC for gene expression quantification in pan-cancer classification.

**Fig. 8:**
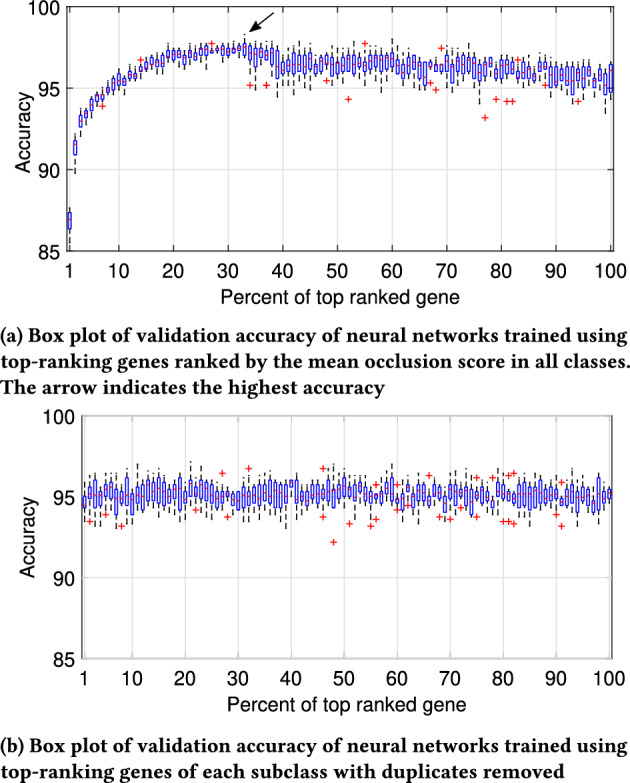
Comparison of validation accuracy of neural network using different gene selection strategy

As demonstrated in Fig. 8, sorting genes based on their mean occlusion scores led to a notable enhancement in validation accuracy, reaching 98.30% with only the top-ranking 33% genes (Fig. 8a). Additionally, two example confusion matrices are provided in Supplementary Fig. 1. Moreover, by constructing a training dataset specific to top-ranking genes sorted by their occlusion scores in each class, we achieved validation accuracies ranging between 94% and 96% (Fig.  8b). These findings underscore the efficacy of our proposed framework in enhancing pan-cancer classification accuracy while reducing the gene count necessary for precise classification.

### Performance comparisons

#### Pan-cancer classification

In this paper, LSTM achieved the best performance in validation accuracy and prediction classes compared to previously reported works on pan-cancer classification. As shown in Table 1, Mostavi et al. achieved an accuracy of 92.5% by implementing various CNN networks featuring a vector of 7100 selected genes and a reshaped 100 $\times$ 71 matrix as input [20]. Similarly, Zhao et al. constructed a network featuring 1D inception architecture and achieved an accuracy of 92.89%, while de Guia et al. and Khalifa et al. converted the gene expression vector into a matrix for CNN training and obtained accuracies of 95.65% and 96.90% [21–23].

**Table 1:** Performance comparison with existing methods

Method	Number of classes	Accuracy	Precision	Recall	F1-score	Comment
LSTM (full gene list)	22 + 6	94.03	85.67	83.02	84.32	22 paired classes + 6 unpaired
LSTM (Top 33% gene)	22 + 6	97.59	97.55	94.63	96.07	22 paired classes + 6 unpaired
Zhao et al.	32	92.89	95.55	95.25	95.40	32 tumor types
Mostavi et al.	33 + 1	95.70	93.60	91.90	92.74	33 tumor types + 1 normal
de Guia et al.	33	95.65	95.55	95.69	95.62	34 tumor types
Sun et al.	10	96.70	99.30	96.90	98.09	11 tumor types
Khalifa et al.	5	96.90	94.96	95.09	95.02	5 tumor types

Additionally, compared to the CNN-based methods, our LSTM neural network can distinguish 28 classes of various tissues of origin and status, which is significantly higher than others. For example, Sun et al. implemented a model to distinguish normal samples and tumor samples without tissue of origin (binary classification) and achieved an accuracy of 96.0%, while their second model for classifying 11 tumor classes with tissue of origin achieved an accuracy of 98.6% [24]. Similarly, Khalifa et al. achieved an accuracy of 96.9% with five tumor classes and no normal class [22].

#### Metastasized cancer classification

To compare the classification performance against Sun et al., metastasized colorectal cancer samples from Kim et al. were utilized as a test dataset for direct comparison [25, 26]. Notably, our accuracy is significantly higher than the reported work using the same dataset. Our study achieved an accuracy of 88.89%, with 16 out of 18 metastasized colorectal cancer samples correctly classified as “colorectal cancer”. The remaining two samples were classified as “colorectal normal” and “liver normal”, respectively.

### Identification of marker gene

#### Marker gene selection method comparison

Selecting appropriate marker genes is crucial in improving pan-cancer classification performance. We compared the performance of several published marker gene selection methods on the same LSTM network, using marker genes selected according to their original method. Additionally, we compared the occlusion score and tissue specificity entropy score and found that the occlusion method is superior to the entropy method in identifying pan-cancer marker genes.

In the study conducted by Mostavi et al., a fixed threshold was used to select genes with an FPKM mean or standard deviation above the threshold [20]. However, as this method has two criteria, it is difficult to control the number of selected genes and thus remains unchanged. Consequently, a gene list containing 29,777 genes was used for preparing the LSTM neural network dataset. In the work done by Zhao et.al’s study, the top 40 genes with the highest difference between the median expression of each gene in the in-class sample relative to the out-of-class samples were selected [23]. A total of 1120 genes were selected using this method. To make it comparable to the occlusion method, the scope was widened to increase the number of marker genes selected.

#### Classification performance comparison

**Fig. 9:**
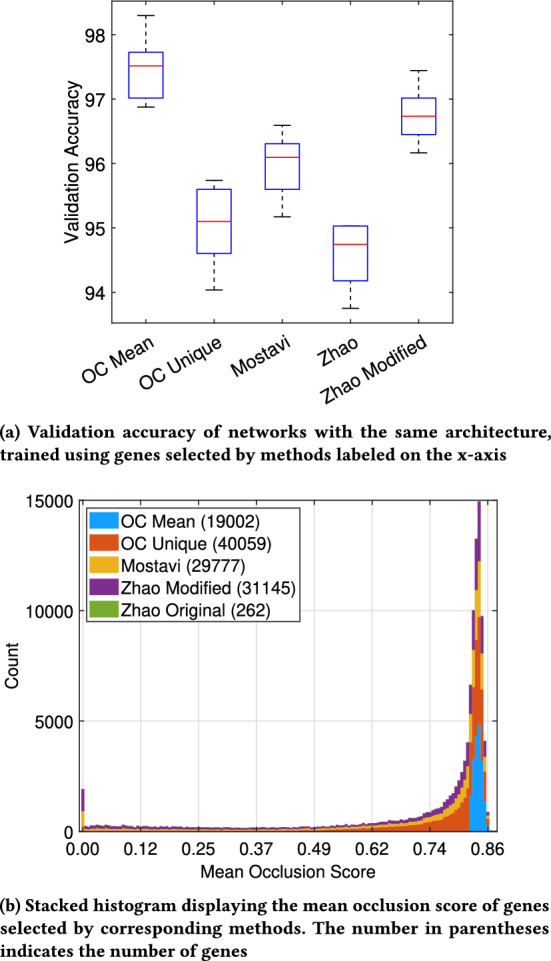
Comparison of the validation accuracy of neural networks trained using genes selected by various methods and their distribution of mean occlusion scores

To compare the performance of different marker gene selection strategies, we trained separate LSTM neural networks with the same architecture using the selected marker genes and applied a fivefold cross-validation strategy. As shown in Fig. 9a, the LSTM network trained with a dataset containing genes selected by the occlusion method achieved the highest median validation accuracy of 97.51% with the fewest number of genes. Meanwhile, the LSTM network trained using a modified selection strategy by selecting the top 33% unique genes from individual 28 classes according to their occlusion score (occlusion unique) attained a median validation accuracy of 95.10%. In comparison, the LSTM network trained with genes selected by Mostavi’s method obtained a median validation accuracy of 96.09%. The original method from Zhao et al. had the lowest median validation accuracy of 94.74%, which increased to 96.73% after widening the criteria to include a similar number of genes as other methods. Further analysis suggests that the mean occlusion score of the genes is an important factor in determining the validation accuracy of the LSTM network.

To investigate the relationship between genes selected by different strategies and the performance of the neural network, their mean occlusion scores are visualized in Fig. 9a for comparison.

*Occlusion Mean:* 19,002 genes selected by their mean occlusion score showed a concentrated right-skewed distribution in the histogram and thus exhibited the best classification performance.

*Occlusion Unique:* The modified occlusion method selects 40,059 unique genes, which is the greatest number of genes, however, the histogram distribution is wider than both the original occlusion method and Mostavi’s method, resulting in lower classification performance.

*Mostavi’s:* Mostavi’s method selects 29,777 genes with a distribution similar to “occlusion unique” but fewer genes with mean occlusion score lower than 130, and thus outperforms “occlusion unique”.

*Zhao’s Modified:* Since the original method from Zhao et al. selected only 262 genes and obtained the lowest performance, the criteria are widened to select 31,145 genes for a fair comparison with other methods. Compared with Mostavi’s method, it is more biased towards genes with higher mean occlusion scores and outperforms Mostavi’s method. It is worth noting that both methods contain genes with the lowest mean occlusion score which hampers the classification performance.

In summary, the mean occlusion score of the selected genes has a significant impact on the neural network’s accuracy; genes with a mean occlusion score higher than 134 improve the accuracy of the network while genes with a lower mean occlusion score have a detrimental effect.

### Absence of correlation between tissue specificity score and mean occlusion score

Genes expressed exclusively in specific cancer types or tissues, and not in others, often exhibit high tissue specificity and may serve as markers for cancer. To quantify this specificity, Schug et al. introduced a tissue specificity score based on Shannon entropy, a concept commonly employed in information theory [27]. Later, an enhanced method known as “ROKU entropy” was published, which further refined the sequencing of genes according to tissue specificity across tissues [28]. To examine whether the occlusion score in this study correlates with these entropies, tissue specificity scores based on Shannon entropy and ROKU entropy were obtained using the software “TSPEX” [29]. The maximum entropy value of Shannon entropy and ROKU entropy output by TSPEX is $log_2N \approx 8.43$ (N = 28), indicating complete tissue specificity. The gene expression matrix for all 28 classes is constructed using the median of FPKM for all samples, as required by the software.

**Fig. 10:**
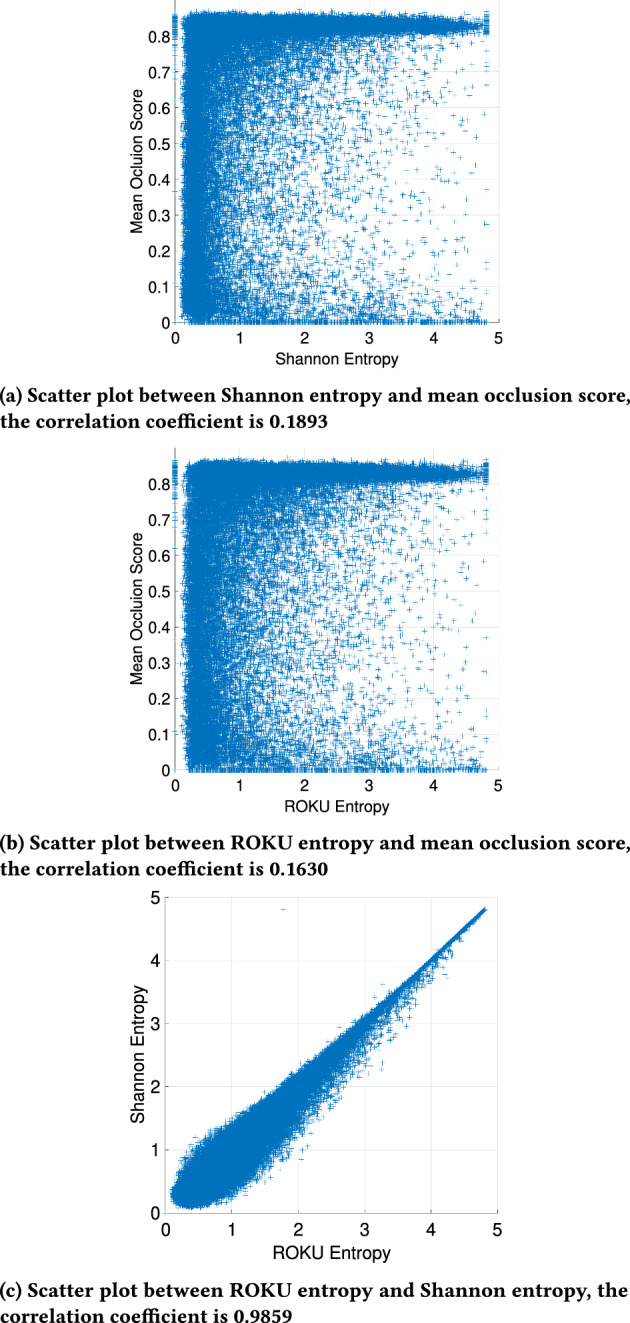
Scatter plots of Shannon tissue specificity entropy score, ROKU entropy score and mean occlusion score

As shown in Fig. 10, there is only a weak positive correlation between Shannon entropy and mean occlusion score (Fig. 10) and a weak positive correlation between ROKU entropy and occlusion score (Fig. 10b. Meanwhile, there is a strong positive correlation between Shannon entropy and ROKU entropy (Fig. 10c). This suggests that, despite lower tissue specificity, some genes are still considered important by the occlusion method. Most importantly, this indicates that the occlusion method evaluates the importance of each gene in pan-cancer classification beyond its tissue specificity.

*Low Tissue Specificity but High Mean Occlusion Score.* To investigate the disparity between tissue specificity score and mean occlusion score, it’s crucial to understand why some genes are not ideal markers for pan-cancer classification despite their high tissue specificity. Investigations were first conducted on genes with a high tissue specificity score but a low mean occlusion score. As shown in Table 2, the gene GFAP is a classical marker of astrocytoma and is exclusively expressed in brain tissue [30, 31]. Therefore, GFAP exhibits one of the highest tissue specificity scores. However, this extremely high tissue specificity also prevents GFAP from being an ideal pan-cancer classification marker, resulting in very low mean occlusion scores. A similar phenomenon was observed with genes such as KRT4, LIPF, TG, and PGC, which are also almost exclusively expressed in specific tissues, making them less suitable for pan-cancer classification [30, 32–34].

**Table 2:** Example genes with low tissue specificity score but low mean occlusion score

Gene ID	Gene name	Mean occlusion score	Shannon entropy	Roku entropy	Tissue specificity
ENSG00000170477.13	KRT4	0.0016	4.65	4.65	Enriched in stomach
ENSG00000096088.16	PGC	0.0011	3.51	3.51	Enriched in stomach
ENSG00000131095.13	GFAP	0.0010	4.78	4.79	Enriched in brain

#### High tissue specificity and high mean occlusion score

Investigations were also conducted on genes with high tissue specificity and a high mean occlusion score. As shown in Table 3, the microRNA MIR663B exhibits both a high mean occlusion score and a high tissue specificity score. Upon closer examination, it is evident that while MIR663B is expressed in several tissues, its expression level in specific tissues such as the brain is significantly higher than in others [35–37].

**Table 3:** Example genes with high tissue specificity score and high mean occlusion score

Gene ID	Gene name	Mean occlusion score	Shannon entropy	Roku entropy
ENSG00000221288.1	MIR663B	0.868	4.807	4.807
ENSG00000199204.1	Y_RNA	0.867	4.807	4.807
ENSG00000230142.2	LINC01075	0.860	4.807	4.807

#### High tissue specificity but low mean occlusion score

In contrast, Table 4 illustrates genes with a high mean occlusion score but a low tissue specificity score. This discrepancy arises from their broad expression across multiple tissues coupled with a lower standard deviation of expression levels. Despite their low tissue specificity, some research has indicated their association with cancer. For instance, the lncRNA ADPGK-AS1 is linked to osteosarcoma, colorectal cancer, pancreatic cancer, and breast cancer through various pathways [38–41]. Another example is the lncRNA DNM1P35, which serves as a novel prognostic factor for kidney cancer [42]. Additionally, STK24-AS1 has been implicated in predicting patient survival rates in colon cancer patients [43].

**Table 4:** Example genes with high mean occlusion score but low tissue specificity score

Gene ID	Gene name	Mean occlusion score	Shannon entropy	Roku entropy
ENSG00000224418.1	STK24-AS1	0.8595	0.2365	0.4062
ENSG00000246877.1	DNM1P35	0.8560	0.3091	0.5346
ENSG00000260898.6	ADPGK-AS1	0.8535	0.2941	0.4468

These findings highlight the superiority of the symmetrical occlusion method over tissue specificity methods in identifying optimal pan-cancer marker genes. Tissue specificity methods, akin to other statistical techniques, may overlook genes with subtle expression level differences. For the complete table, please refer to Supplementary File 2.

### Literature search on top marker genes

To demonstrate the clinical significance of our findings and the relevance of marker genes with the highest occlusion scores to cancer, literature searches were conducted on ZNF709 (protein coding), MIR663B (MicroRNA), and FGF14-AS2 (lncRNA) as examples.

#### ZNF709

Zinc Finger Protein 709 (ZNF709) is a protein-coding gene belonging to the zinc finger family, involved in cellular processes like transcriptional regulation, DNA repair, and cell differentiation [44–46]. Despite its low tissue specificity score (Shannon entropy: 0.3253, ROKU entropy: 0.4742), it stands out as the 4th gene with the highest mean occlusion score. The Human Protein Atlas reports strong expression of ZNF709 in cancers such as thyroid, colorectal, and breast cancer [47, 48].

Heyliger et al. discussed the clinical relevance of ZNF709 in clear cell renal carcinoma, suggesting its downregulation is associated with significantly favorable survival outcomes [49]. Wang et al. identified ZNF709 as one of the independent prognostic factors for pancreatic cancer [50]. Knockdown studies conducted by Yan et al. showed that downregulation of ZNF709 led to increased expression of p53, a well-known therapeutic target for cancer treatment [51–53].

Therefore, ZNF709 could potentially be a target for therapeutic intervention, where increasing its expression levels might enhance the tumor suppressor functions of p53.

#### MIR663B

MicroRNA 663B (MIR663B) is a small RNA molecule involved in gene regulation, specifically as part of the microRNA family. MicroRNAs play a crucial role in post-transcriptional regulation by binding to target messenger RNA molecules, thereby modulating their stability and translation. MIR663B exhibits very high tissue specificity scores (Shannon entropy: 4.8074, ROKU entropy: 4.8074) and is ranked 7th by mean occlusion score.

Recent studies have highlighted the potential significance of MIR663B in cancer progression and treatment. For instance, Jiang et al. elucidated the role of MIR663B in tamoxifen resistance in breast cancer, suggesting its involvement in modulating TP73 expression, a key factor in drug resistance mechanisms [54, 55]. Wang et al. demonstrated that MIR663B promotes cell proliferation and epithelial-mesenchymal transition in nasopharyngeal carcinoma by directly targeting SMAD7 [56]. Additionally, You et al. found that MIR663B exposed to TGF-$\beta$1 promotes cervical cancer metastasis and epithelial-mesenchymal transition by targeting MGAT3 [57]. Guo et al. also revealed that MIR663B targets GAB2 to restrict cell proliferation and invasion in hepatocellular carcinoma [58].

#### FGF14-AS2

Fibroblast Growth Factor 14 Antisense RNA 2 (FGF14-AS2) is a long non-coding RNA involved in gene regulation, particularly in post-transcriptional regulation by binding to target messenger RNA molecules and modulating their stability and translation. Despite its low tissue specificity scores (Shannon entropy: 0.6033, ROKU entropy: 0.9991), FGF14-AS2 ranks 61st (top 1%) in terms of mean occlusion score.

Experimental studies by Yang et al. and Jin et al. have explored the function of long non-coding RNA FGF14-AS2 in breast cancer, revealing its role in repressing metastasis and suggesting its potential therapeutic implications [59, 60]. Additionally, Hou et al. elucidated the inhibitory effect of FGF14-AS2 overexpression on colorectal cancer proliferation via the RERG/Ras/ERK signaling pathway by sponging microRNA-1288-3p [61]. Moreover, Li et al. demonstrated that FGF14-AS2 inhibits prostate carcinoma cell growth by modulating the miR-96-5p/AJAP1 axis, indicating its tumor-suppressive role in prostate cancer [62].

### Marker gene indentification in single cell RNA-Seq

To further verify whether the symmentrical occlusion could accurately classify cell types, a separate LSTM neural network was trained on human muscle “single-cell RNA Sequencing” (scRNA-Seq) data acquired from public sources using the same workflow. The neural network obtained a validation accuracy of 96.0%, indicating that the network can accurately identify cell types using scRNA-Seq data.

#### Loss of function simulation

To verify that the symmentrical occlusion is able to identify known marker genes, 100 randomly selected “Muscle Stem Cells” (MuSC) cells were extracted from the validation dataset. Then, the expression level of the gene PAX7 which is one of the marker genes of MuSC cells and only expressed in MuSC, was reduced to simulate “loss of function”.

**Fig. 11:**
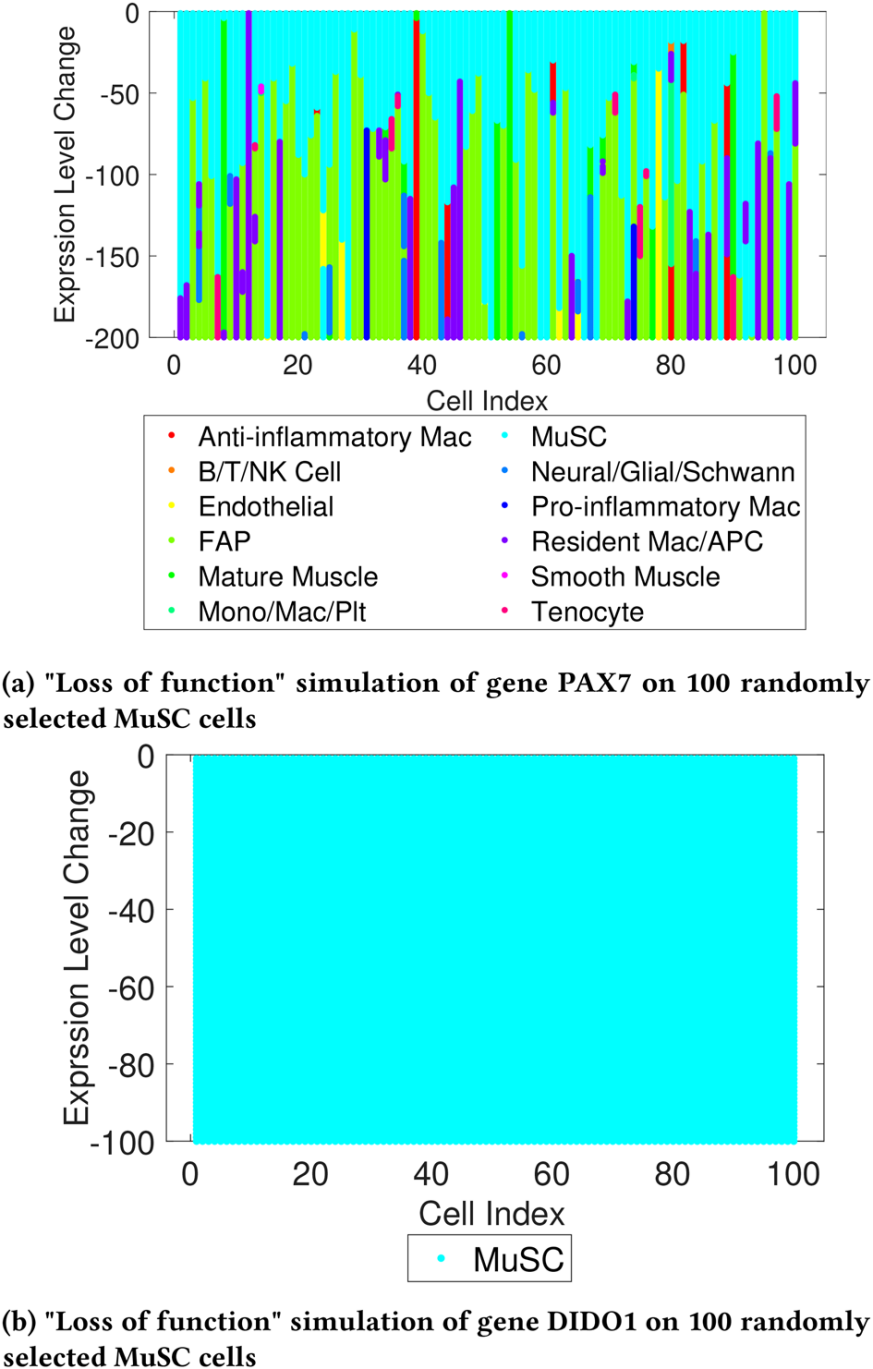
“Loss of function” simulation on known marker gene of MuSC cells and randomly selected gene in MuSC cells

As shown in Fig. 11a, from top to bottom, as the expression level of gene PAX7 decreases during the occlusion process, many of these cells begin to be misclassified as non-MuSC, indicating that PAX7 is indeed an essential gene for the identification of MuSC cells. Meanwhile, Fig. 11b shows that reducing the expression level of the gene Dido1 which was randomly selected, had no impact on the prediction result of another 100 randomly selected MuSC cells.

#### Gain of function simulation

**Fig. 12:**
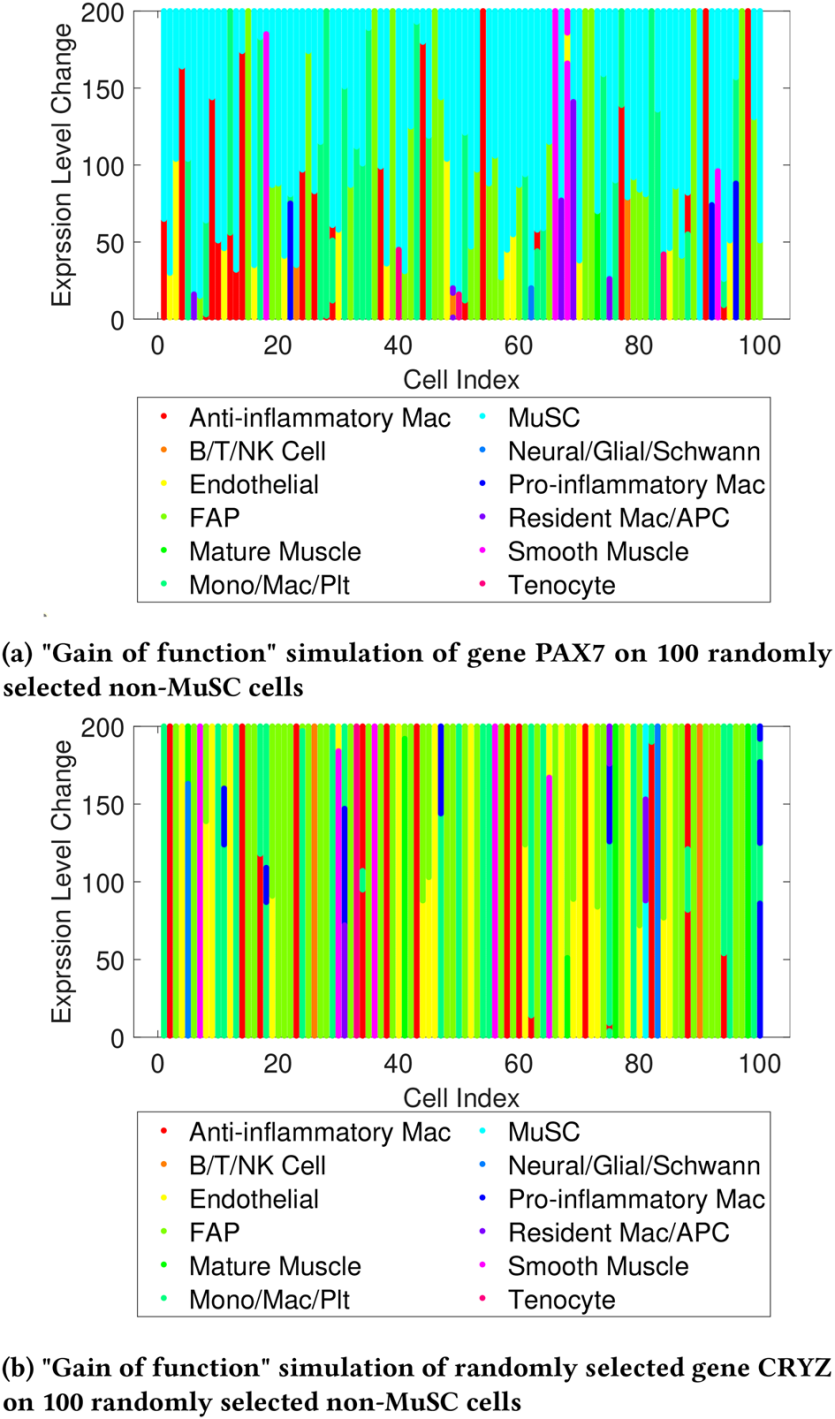
“Gain of function” simulation on known marker gene of MuSC cells and randomly selected gene in non-MuSC cells

To underscore the significance of PAX7 as a key marker gene for MuSC cells, the expression level of PAX7 was increased in 100 randomly selected non-MuSC cells to simulate the “gain of function” of PAX7. As shown in Fig. 12a, from bottom to top, the classification results reveal a notable shift, with the majority of non-MuSC cells being classified as “MuSC” as the expression level of PAX7 is systematically increased.

In stark contrast, as shown in Fig. 12b increasing the expression level of the randomly selected gene, CRYZ, in 100 non-MuSC cells had little to no impact on the classification result. This stark difference in outcomes emphasizes the influential role that gene PAX7 plays in the accurate identification of MuSC cells compared to randomly selected genes.

This result highlights the potential of the LSTM neural network for cell type classification using scRNA-Seq data. Moreover, by conducting symmetrical occlusion on both marker genes of MuSC and non-MuSC cells, we have demonstrated the effectiveness of the occlusion method in identifying marker genes. These findings may contribute significantly to the advancement of more accurate and efficient methods for single-cell classification and annotation. The robust performance of the LSTM network and the insights gained from symmetrical occlusion pave the way for enhanced methodologies in the field, offering valuable tools for researchers working with scRNA-Seq data to unravel the complexities of cellular heterogeneity.

### Gene ontology analysis on identified marker genes

**Fig. 13:**
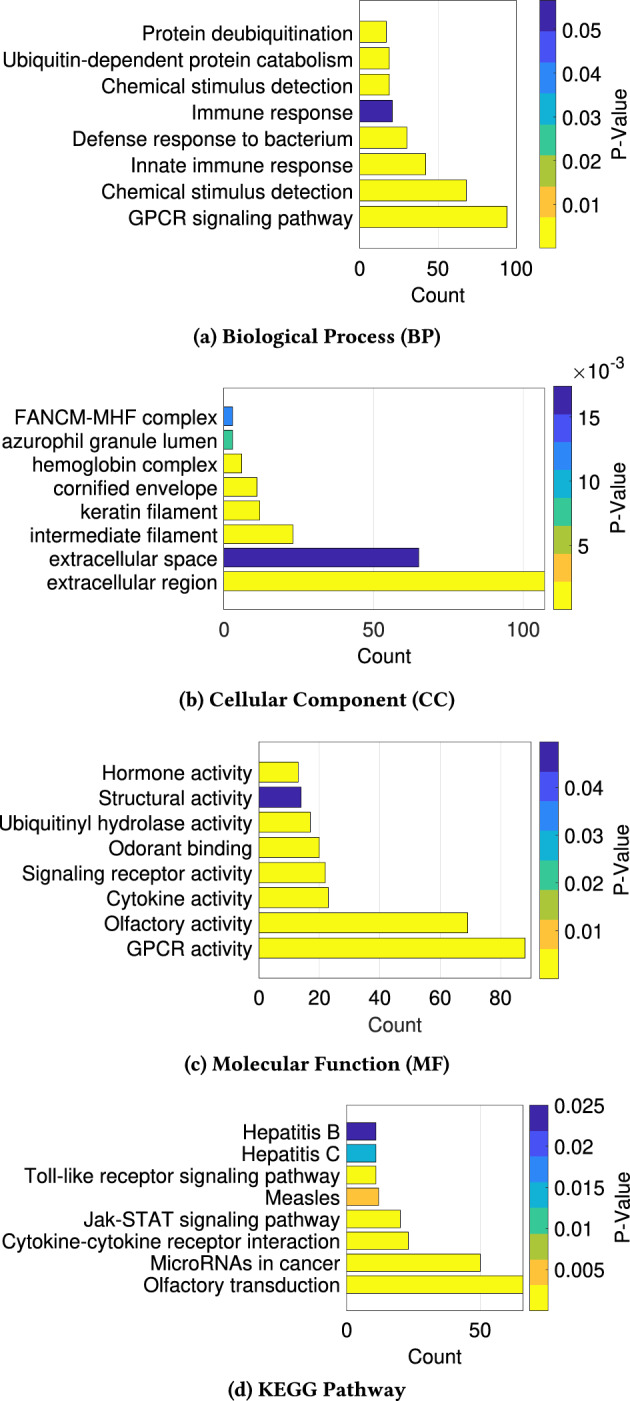
Gene ontology analysis result on top 33 percent genes with the highest mean occlusion score

The top 33% of genes, ranked by occlusion score, were subjected to “Gene Ontology” (GO) term analysis using DAVID to confirm their relevance to cancer [63]. As shown in Fig. 13, the most significant molecular function identified is “olfactory receptor activity”, which has been linked to the perception of smell and cancer by prior publications. For instance, Shibel et al. reported that olfactory receptor OR5H2 regulates the proliferation of endometrial cancer cells through the IGF1 signaling pathway [64].

Furthermore, PSGR, a prostate-specific G protein-coupled receptor, has been found to be upregulated in prostate cancer. Another study by Webber et al. reported that olfactory receptor OR10H1 is primarily expressed in human bladder cancer [65]. Pathway analysis also revealed olfactory transduction to be the most significant pathway, followed by “micro RNAs in cancer” and JAK-STAT signaling. The JAK-STAT pathway is known to promote tumour genesis, and its inhibition can impede cancer cell growth [66]. Overall, these findings suggest that the selected genes may be implicated in cancer-related processes. The complete result can be found in Supplementary File 3.

### Pseudogenes among the top-ranking genes

Pseudogenes were once considered non-functional copies of parental genes resulting from mRNA retrotransposition or genomic duplication, seemingly lacking biological significance. However, recent research has unveiled their diverse roles in physiological and pathological processes, particularly in cancer contexts [67]. Studies indicate that pseudogene expression patterns vary across tumor subtypes and can even impact patient survival in specific cancers like kidney cancer [68–70].

Among the top 33% of genes ranked by mean occlusion score, 6,108 are pseudogenes. To explore any correlation with previous findings, 2,616 pseudogenes reported by Han et al. as differentially expressed across four tumor types were selected. Corresponding ensemble gene IDs were extracted via the ensemble.org REST API and cross-referenced to ensure consistency in genomic location. Of these, 974 pseudogenes had valid gene IDs, with significant representation from different tumor types: 34.85% from Glioblastoma (GBM), 27.76% from Breast Cancer (BRCA), 27.67% from Lung Squamous Cell Carcinoma (LUSC), and 10.47% from Uterine Corpus Endometrial Carcinoma (UCEC), all within the top 33% of genes selected by the occlusion method. This suggests that pseudogenes may significantly contribute to pan-cancer classification.

To gauge the contribution of pseudogenes, a depletion study was conducted. LSTM neural networks were trained using updated datasets with pseudogenes excluded from the top 33% of genes. Surprisingly, networks trained without pseudogenes exhibited a marginal decrease in average accuracy (0.5%) compared to networks trained with pseudogenes included. This indicates that while pseudogenes may have a limited impact on pan-cancer classification accuracy, their inclusion remains crucial for optimal performance.

## Discussion

This paper showcases the effectiveness of the NRC normalization method and the LSTM neural network in pan-cancer classification and marker gene prediction using both RNASeq and scRNA-Seq data. Additionally, the proposed occlusion algorithm proves its efficacy in identifying marker genes and enhancing the classification accuracy of the neural network with fewer genes.

Moreover, the occlusion algorithm has the potential to unveil gene-gene interactions by testing combinations of candidate genes, albeit this approach may pose computational challenges due to the vast number of possible combinations. These findings underscore the versatility and promise of the occlusion algorithm for diverse applications in genomics research.

Beyond cell type classification using scRNA-Seq data, the LSTM neural network shows promise in detecting novel cell types absent from the training dataset. This is evidenced by the network’s tendency to misclassify novel cell types with low prediction scores. For instance, when MuSC cells are excluded from the training dataset but included for prediction, the network misclassifies all MuSC cells as other cell types, yet with a markedly lower prediction score (0.4–0.6) compared to true positive results (approximately 1). Leveraging this discrepancy enables the detection of novel cell types, achieving an accuracy of 85–88% when employing machine learning classifiers such as linear discriminant and SVM.

### Supplementary Information

Supplementary Material 1.

Supplementary Material 2.

Supplementary Material 3.

Supplementary Material 4.

## Data Availability

All data generated or analyzed during this study are publicly available using their accession numbers in the supplementary files. The code is available from the corresponding author upon reasonable request.
