# Supplementary material for: Occlusion enhanced pan-cancer classification via deep learning
Source: BMC Bioinformatics. 2024 Aug 8;25:260. doi: 10.1186/s12859-024-05870-y (PMC11308240; doi:10.1186/s12859-024-05870-y)
Supplement: Supplementary file 1 — Supplementary Material 1. [file 12859_2024_5870_MOESM1_ESM.pdf]

A

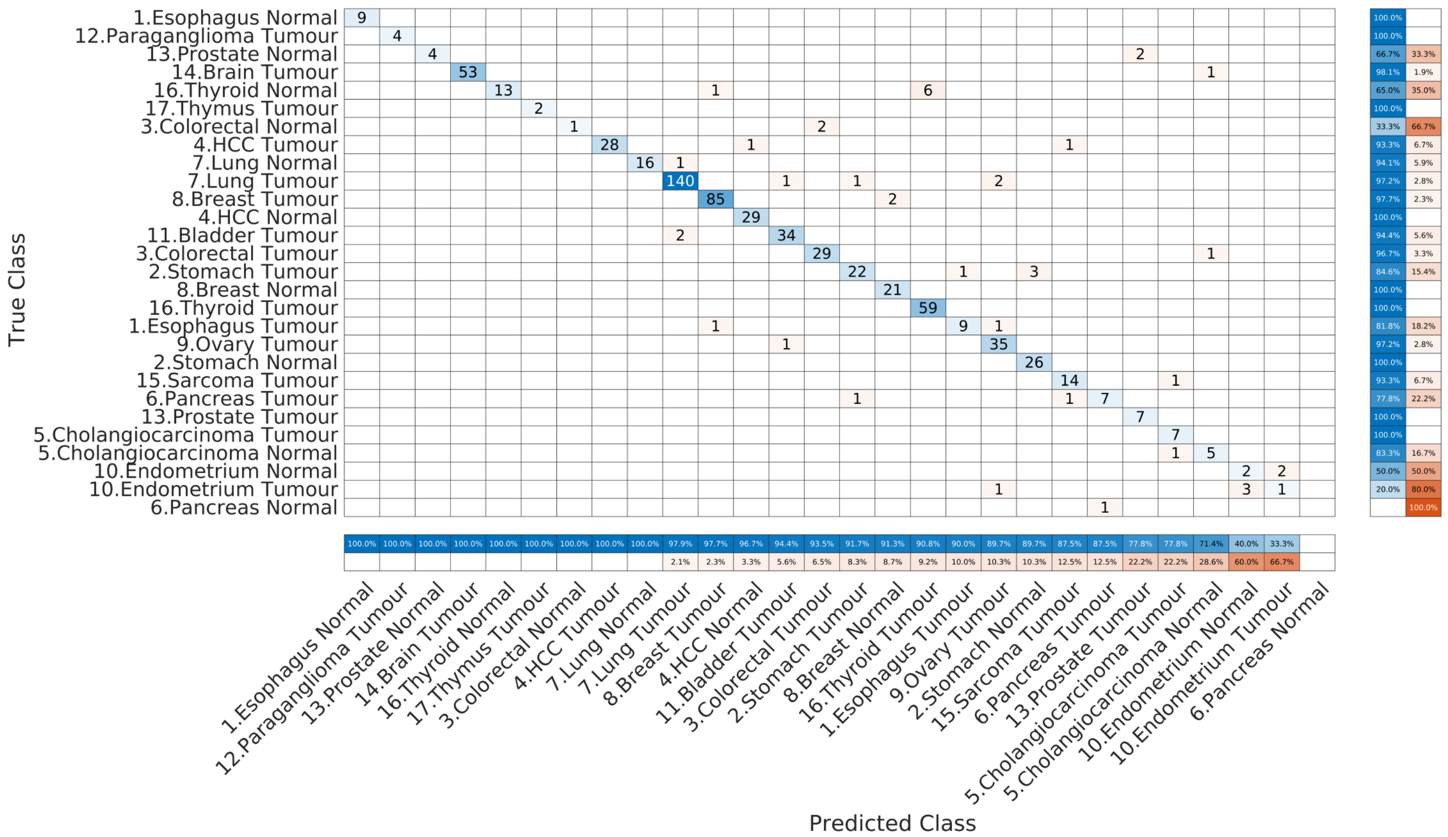

B

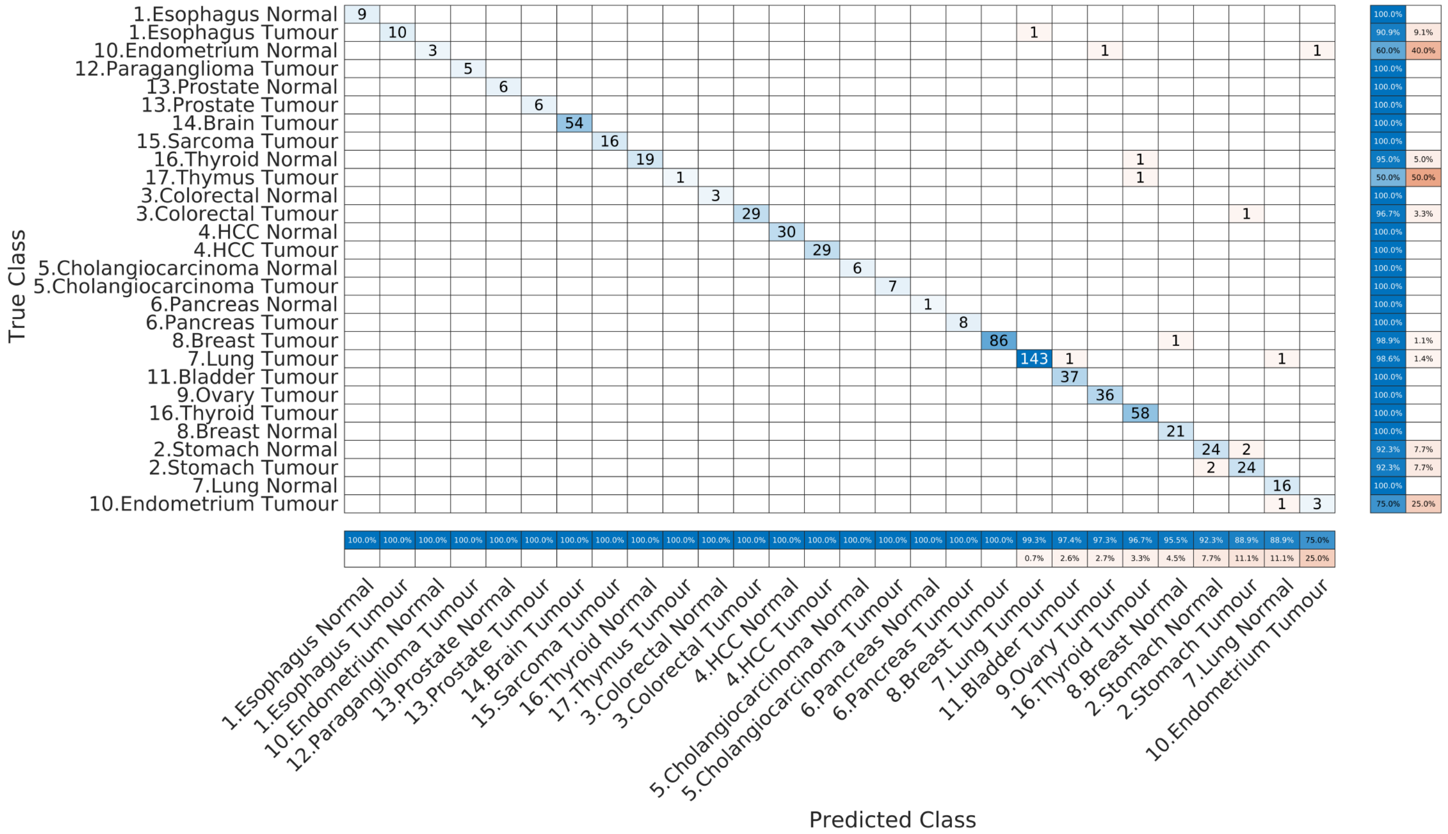

Supplementary Fig.1 Confusion matrices comparison before and after optimization using occlusion, sorted by precision. (A) Confusion matrix of neural network trained using all genes. **(B)** Confusion matrix obtained after selecting top 33% genes with highest mean occlusion score in all classes and trained new neural network. After optimization, the new neural network has better performance with fewer genes. The only difference between the two is the adjustment in input layer as the size of input has drastically shortened.
